# 19 patients report seizure freedom with medical cannabis oil treatment for drug-resistant epilepsy: a case series

**DOI:** 10.3389/fnins.2025.1570531

**Published:** 2025-05-19

**Authors:** Frank Yizhao Chen, Joshua Myles Duckman, Brenden Samuel Rabinovitch, Katrin Julia Hannesson, Evan Cole Lewis

**Affiliations:** ^1^North Toronto Neurology, Toronto, ON, Canada; ^2^Department of Research, JMCC Group, Toronto, ON, Canada; ^3^Division of Experimental and Translational Neuroscience, Krembil Brain Institute, University Health Network, Toronto, ON, Canada; ^4^Department of Physiology, Temerty Faculty of Medicine, University of Toronto, Toronto, ON, Canada; ^5^Department of Pediatrics, Temerty Faculty of Medicine, University of Toronto, Toronto, ON, Canada

**Keywords:** epilepsy, cannabis, CBD–cannabidiol, THC–tetrahydrocannabinol, seizure freedom, pediatric epilepsy, seizures, cannabis medicine

## Abstract

**Purpose:**

Seizure freedom (SF) is the primary goal of epilepsy treatment. More treatments that produce SF in drug-resistant epilepsy (DRE) are needed. Cannabis-based products for medicinal use (CBPMs) containing cannabidiol (CBD) and Δ9-tetrahydrocannabinol (THC), administered as oils, have been shown to induce SF in DRE. However, there remains a paucity of published real-world evidence in both pediatrics and adults on SF resulting from CBPM therapy.

**Methods:**

This is a retrospective case series at an outpatient neurology clinic in Toronto, Canada, on patients with DRE who experienced significant SF during CBPM treatment. All patients were treated via the clinic’s stepwise treatment protocol with CBPM oils only. The study describes clinical features of patients and their CBPM-related SF.

**Results:**

We report 19 DRE cases that experienced SF; 15 pediatric, 4 adults. The median cumulative SF duration was 245 days, split between continuous SF periods lasting at least 90 days. Five patients had continuous SF periods lasting ≥ 1 year. Most patients used CBD+THC regimens. Three patients weaned all concomitant ASMs. Adverse events (AEs) were reported by half of the patients.

**Conclusion:**

The results of the study support prioritizing CBPMs in cases of DRE. It also supports research into identifying clinical and biological biomarkers for DRE cases that may achieve SF under CBPM treatment. Lastly, the study supports improving the accessibility of CBPMs, using SF as a primary outcome in future CBPM epilepsy trials, and assessing the role of THC in reducing seizures.

## Introduction

Epilepsy is characterized by recurring, spontaneous seizures affecting > 1% of individuals worldwide (GBD 2016 Epilepsy Collaborators, 2019). Poor seizure control has harmful consequences, including impaired quality of life (QoL), high rates of psychiatric comorbidities and mortality, (Mohammadzadeh and Nazarbaghi, 2022) increased financial burden, and a high likelihood of cognitive impairments (Laxer et al., 2016). Conversely, people with epilepsy (PWE) achieving seizure freedom (SF) report significant QoL improvements (Jain et al., 2020) even when compared to PWE with just one seizure within the past 5 years (Josephson et al., 2017). Patients and caretakers also rank SF as their primary treatment outcome (Josephson et al., 2017; Halford and Edwards, 2020).

Medical intervention with anti-seizure medications (ASMs) is the first line of treatment to reduce seizures. Most ASMs modulate neuronal excitability by targeting ion channels and neurotransmitters (Manford, 2017). For example, carbamazepine (CZP) and valproic acid (VPA) are inhibitors of voltage-gated sodium and L-type voltage-gated calcium channels, preventing action potential propagation in neurons (Gambeta et al., 2020). VPA also has a bipartite mechanism on GABA, that works to both reduce the breakdown of and increase the release of GABA, thus shunting spreading depolarization and seizures (Rahman et al., 2025). Though ion-channel based mechanisms like these are appropriate for some seizures, there are few alternative ASMs that operate on separate mechanisms that may be better suited for certain epilepsies, as reflected in the current rates of ASM-induced SF. While 37% (Brodie et al., 2012) to 50.5% (Chen et al., 2018) of patients experience significant SF (i.e., > 1 year) after starting their first ASM, the probability of SF decreases substantially with each successive ASM regimen (Brodie et al., 2012). Chen et al. (2018) found that the second and third regimens provide just an additional 11.6% and 4.4% likelihood of SF, respectively, and that there was a 1.73-fold increase in the probability of uncontrolled seizures occurring with each successive ASM treatment. Further, with about 50% of epilepsy cases having no identifiable etiology (Thijs et al., 2019), ASM selection is challenging and, at times, based on clinical features or practitioner experience alone, rendering SF a difficult outcome to achieve. Thus, many PWE try several different ASMs, which is associated with economic burden (de Kinderen et al., 2014), reduced QoL (Puri et al., 2018), and disruptive ASM-related adverse events (AEs) (Perucca et al., 2018), without reaching SF.

A diagnosis of drug-resistant epilepsy (DRE) is made when a patient fails to respond to more than two appropriately trialed ASMs (Sultana et al., 2021). More than 30% of PWE suffer from DRE (Tang et al., 2017), and its associated increases in risk of comorbidities, number of hospitalizations, and mortality rates compared to non-DRE PWE (Strzelczyk et al., 2017). At least one third of individuals with DRE have a psychiatric comorbidity. The risk of depression is 2.7-fold greater in individuals with epilepsy than in the general population. Psychiatric comorbidities, including depression (major depression and treatment-resistant depression), anxiety/anxiety-related disorders, and functional seizures (psychogenic non-epileptic seizures, PNES) have disproportionate prevalence in the epilepsy population, reaching rates of 23.1, 20.1, and 9–12%, respectively (Mula et al., 2021).

In support of the definition of DRE, Chen et al. (2018) analysis of their DRE cohort of 1,792 pediatric and adult patients demonstrated that, beyond the third ASM, the chance of achieving SF in this population was 1% or less and “cumulative probabilities of seizure freedom [after 2 drug trials] were not significantly different with each successive” ASM. These outcomes have not improved compared to similar research from over two decades ago, indicating a need for a “paradigm shift in treatment and research strategies” (Chen et al., 2018). Non-pharmaceutical options exist for DRE, but these vary in efficacy; have inherent accessibility barriers; and pose independent, significant risks (Solli et al., 2020). Thus, exploring alternative treatments that achieve SF is necessary.

A promising treatment for DRE are cannabis-based products for medicinal use (CBPMs) containing variations of cannabinoids, such as cannabidiol (CBD), Δ-9-tetrahydrocannabinol (THC) and other cannabis plant products in an oil formulation. Cannabinoids act by binding to receptors to modulate the endocannabinoid system, contrasting the mechanisms by which most ASMs operate. Pre-clinical work established CBD’s anti-seizure properties (Jones et al., 2010) while clinical randomized control trials of purified CBD demonstrated effective seizure reduction in patients with Dravet syndrome (Devinsky et al., 2011; Devinsky et al., 2018b; Miller et al., 2020), Lennox-Gastaut syndrome (French et al., 2017; Devinsky et al., 2018a; Thiele et al., 2018), and Tuberous Sclerosis complex (Thiele et al., 2021), with 50% seizure response rates in refractory epilepsy ranging from approximately 35% to 63% (Hausman-Kedem et al., 2018; Lattanzi et al., 2018; McCoy et al., 2018; Devinsky et al., 2019; Devinsky et al., 2020; Lattanzi et al., 2020a; Thiele et al., 2022). These trials led to United States Food and Drug Administration, European Medical Association, and Health Canada approvals for Epidiolex^®^—a purified CBD product—for DRE in patients 2 years and older suffering from seizures due to these conditions (US Food & Drug, 2018; Ema, 2019; Product information, 2024). Collectively, past RCTs and open-label trials of CBD have demonstrated its contribution to SF in approximately 4% of DRE patients (Table 1).

**TABLE 1:** Studies assessing oil-based CBPM treatment of epilepsy that report complete seizure freedom rates and have an *N* > 10, as of October 2023.

Study type	References	Cohort	Treatment	Total final CBPM *n*	Complete seizure freedom rate
RCT	Devinsky et al., 2011	Drug-resistant Dravet syndrome	CBD	60	5.0% (*n* = 3)
	Thiele et al., 2021	Drug-resistant seizures tuberous sclerosis	CBD	126	4.7% (*n* = 6)
	Miller et al., 2020	Dravet syndrome	CBD	124	4.0% (*n* = 5)
RCT aggregate	–	–	–	310	4.5% (*n* = 14)
Open-label	Devinsky et al., 2016	Treatment-resistant epilepsy	CBD	137	1.5% (*n* = 2)
	Hess et al., 2016	DRE tuberous sclerosis	CBD	18	5.6% (*n* = 1)
	Patel et al., 2021	Lennox-Gastaut syndrome	CBD	366	4.6% (*n* = 17)
	Scheffer et al., 2021	Dravet syndrome	CBD	290	4.1% (*n* = 12)
	Thiele et al., 2022	Tuberous sclerosis complex	CBD	199	3.5% (*n* = 7)
	Tzadok et al., 2016 (retrospective)	Intractable pediatric epilepsy	CBD+THC	74	2.7% (*n* = 2)
	Hausman-Kedem et al., 2018	Refractory epilepsy	CBD+THC	46	4.3% (*n* = 2)
Open-label aggregate	–	–	–	1,130	3.8% (*n* = 43)

Preclinical research regarding the effects of THC on seizures has yielded mixed results. In different rodent studies, seizures were induced with high doses of THC (Malyshevskaya et al., 2017; Anderson et al., 2020) while low-dose THC, co-administered with CBD, improved seizure outcomes (Anderson et al., 2020; Dlugosz et al., 2023). Published open-label and observational studies of THC in humans with epilepsy support the latter finding from animal studies (Pamplona et al., 2018). CBD-rich extracts demonstrate similar degrees of seizure reduction compared to purified CBD in patients diagnosed with Dravet syndrome and other epileptic encephalopathies (Pamplona et al., 2018; Huntsman et al., 2019; Zafar et al., 2021) and clinical reports by Nowicki et al. (2022) and Erridge et al. (2023) presented evidence that add-on THC may contribute to achieving SF in pediatric DRE patients (Nowicki et al., 2022; Erridge et al., 2023).

Despite CBPM research presenting meaningful seizure response rates and significant seizure reductions, there is an absence of research on the practical aspects of achieving SF with CBPMs in real-world settings and the qualities that define this special population. For instance, there is no published data on clinical biomarkers predicting SF, clinical protocols associated with achieving SF, or longitudinal clinical outcomes. Every CBPM epilepsy study to date has reported SF rates as a secondary outcome with little to no attention paid to specific features of this special population.

The aim of this study was to focus on this population and report its clinical features to provide a basis for future research. This may help demarcate this population from those with DRE providing predictive clinical metrics to determine the most robust responders to CBPM therapy in the context of DRE. To achieve this, we analyzed the clinical features of 19 patients with DRE treated with CBPMs who experienced relevant and complete seizure-free periods lasting more than 3 months, with a specific emphasis on patients who experienced SF periods lasting at least 1 year.

## Materials and methods

### Study design

This is a retrospective case series using medical records from an outpatient neurology clinic in Toronto, Ontario (North Toronto Neurology [NTN]; formerly Neurology Centre of Toronto). The study included patients that (1) started CBPMs to improve their seizures between 1st January 2018 and 28th February 2023; (2) were supervised by an NTN neurologist with specialization in pediatric epilepsy; (3) had DRE, as defined by the International League Against Epilepsy (ILAE) (Kwan et al., 2010); and (4) experienced at least one continuous and substantial SF period, marked by freedom of all seizure types (i.e., seizure frequency of 0). A continuous and substantial SF period was defined as at least 90 days in which no seizures were experienced and occurring 90 days after the addition/alteration of CBPMs, without altering other treatments. This timeline was established to reduce the potential of confounding from the “honeymoon effect,” which is associated with several ASMs (Avanzini, 2006; Löscher and Schmidt, 2006; Chen et al., 2018) including CBD (Lattanzi et al., 2020b; Uliel-Sibony et al., 2021; Kühne et al., 2023). We performed further analyses on patients with continuous SF periods of at least 1 year, to align with the ILAE’s definition of SF (Kwan et al., 2010). Patients were excluded if (1) they were using CBPMs for non-seizure conditions; (2) their CBPM treatment was not guided by NTN; (3) they were self-medicating with cannabis products, as reported by the patients; or (4) their SF was due to the alteration/addition of a different treatment. Ethical approval for this study was received by Veritas, a Canadian Independent Review Board (Reference number: 2021-2597-5657-2).

### Medical cannabis program

All patients were treated under the NTN Medical Cannabis Program and received authorization to access medical cannabis from the program’s neurologist. The program commences with an education session and an initial consultation with the clinical team. If the patient is medically suitable (no history of unstable/severe cardiac/renal/hepatic impairment; no active/unstable psychiatric condition), they are granted authorization to use CBPMs, adhering to the treatment protocol, and according to the Canadian regulatory framework governing CBPM authorization. The program’s clinical team remained consistent throughout the observation period.

The Medical Cannabis Program treatment protocol for epilepsy is based on published evidence, neurobiological mechanisms, and team-based clinical experience. Its structure aligns with the first principles of epilepsy care, in that CBD and THC are regarded as two distinct ASMs used in combination (Sander, 2004). Prior to commencing treatment, standard and relevant bloodwork and electrocardiogram are ordered. Patient diagnoses and epilepsy evaluations are also confirmed by electroencephalograms (EEGs) prior to commencing treatment. At the initial consultation, the neurologist helps patients parse their seizures into different seizure types. The protocol consists of 5 phases, and follows a step-wise titration of CBD, then THC based upon the patient’s clinical response (Supplementary Figure 1). When THC is added, CBD dosage is reduced. As with management using conventional ASMs, if a patient fails to respond or exhibits only a partial response during each visit, dosing progresses to the next phase. Conversely, if a patient demonstrates an “adequate response,” dosing is maintained. EEGs during and after CBPM treatment were not ordered, due to feasibility issues of a real world study. Adequate response is defined as a change in seizures (frequency, duration or severity) that meets patient goals of care (e.g., seizure freedom/reduction, improved QoL, reduced CBPM/ASM AEs, etc.), and is highly individualized and dynamic which is typical in the DRE population. CBPM progression and discontinuation is weighed against AEs, similar to adding new ASMs to treatment regimens.

Prescribed CBPMs were those available in Ontario, Canada for medical use. They included oil-based isolated cannabinoids or broad/full spectrum. No patients were prescribed dry flower preparations. Epidiolex^®^ (approved in Canada in 2024) was unavailable in Canada during the study period and, therefore, no patients reviewed were using this product.

### Seizure tracking

Seizure counts from baseline, during, and after CBMPs were based on information obtained from the caretaker/patient at each FU. To establish baseline numbers, patients/caregivers are instructed to record at least two weeks of baseline seizure frequency and duration with respect to each seizure type. At each subsequent visit and during CBPM treatment, patients/caregivers are instructed to track seizures. The neurologist reviews each seizure type and respective frequencies and durations at FUs. Patients and caregivers were not given standard seizure calendars or trackers. Instead, patients used a variety of tools, ranging from handwritten calendars to seizure-tracking apps.

### Data collection/analyses

All relevant data were collected from the NTN electronic medical records, which were stored in a TELUS PS Suite^®^ EMR software hosted locally on NTN servers. Baseline EEGs used to affirm preliminary diagnoses were unavailable for analysis. Eligible patients were identified using PS Suite’s built-in query tools, to generate an anonymized list of all patients who underwent CBPM treatment at NTN during the observation period. Manual and automated data cleaning processes were performed to assess eligibility. A further round of data cleaning was performed by the authors (EL and FC) to affirm SF patient eligibility and data accuracy. Data was only available to research and clinical care teams. Missing data was marked in the dataset, but core data was available for all eligible patients. Extracted data included SF information (e.g., length of SF period; SF dosages; AEs) and secondary outcomes (demographics, epilepsy history, CBPM information, seizure/QOL changes at each follow-up [FU], ASM weaning). Seizure changes, QoL, and AE measures were from patient self-reports during appointments. The main goal of this study was to describe SF in the context of CBPMs. Python and its PANDAs library were used to clean the data. All study outcomes were presented using descriptive statistics performed in Microsoft Excel^®^; no inferential statistics were performed.

## Results

### Demographics and epilepsy medical history

**FIGURE 1:**
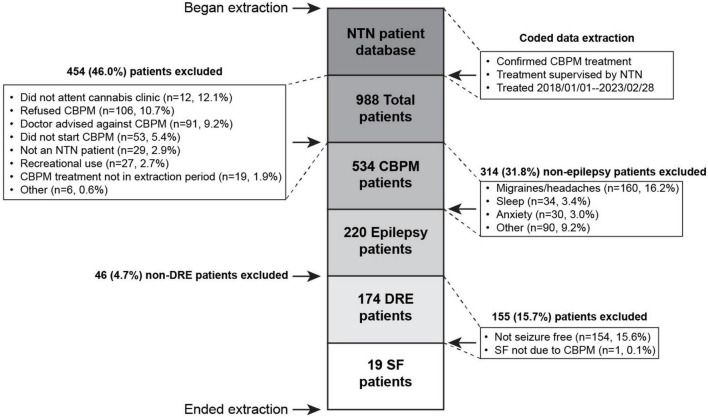
Flow chart of data extraction timeline with inclusion and exclusion criteria.

The extraction process (Figure 1) yielded 174 patients with DRE. From these, 19 (10.9% of initial cohort) DRE patients met the SF criteria for this study. One patient experienced SF while on CBPMs, but had to be excluded because their SF occurred immediately after a change in lamotrigine and valproic acid (VPA) dosing, violating exclusion criterion #4. Pediatric patients comprised 79% (*n* = 15) of SF patients. Table 2 summarizes the demographic information of the SF cohort, while Supplementary Table 1 presents patient-specific demographics. All relevant epilepsy medical history data is summarized in Table 2 and Supplementary Table 1. The most common seizure types were Generalized Tonic-Clonic (*n* = 13, 69%), Absence (*n* = 8, 42%), and Myoclonic seizures (*n* = 3, 16%) (Supplementary Table 1). The cohort represented a wide range of syndromes, seizure types and etiologies. The cohort was treated with a variety of anti-seizure interventions as shown in Table 2 and Supplementary Table 2. During CBPM treatment, 13 patients were taking concomitant ASMs. The median reported seizure frequency per month before CBPMs was 6.50 (range 0.5–12,300), with each patients’ pre-CBPM seizure count reported in Supplementary Table 1. For the full summary of specific CBPM each patient was on, see Supplementary Table 4.

**TABLE 2:** Demographic data on the cohort.

Demographic and medical history	Pediatric (< 18 yo) (*n* = 15)	Adult (*n* = 4) (≥ 18 yo)	Total (*n* = 19)
**Sex**			
Male	*n* = 7 (46.7%)	*n* = 1 (25%)	*n* = 8 (42.1%)
Female	*n* = 8 (53.3%)	*n* = 3 (75%)	*n* = 11 (57.9%)
**Age**			
At seizure onset	Median = 2	Median = 2.85	Median = 2
	IQR = 1–3.5	IQR = 0.66–9.5	IQR = 0.85–4
	Range = 0–9	Range = 0.54–23.0	Range = 0–23
At SF start	Median = 5.08	Median = 27	Median = 7.58
	IQR = 3.91–10.72	IQR = 25.25–30.5	IQR = 4.14–16.81
	Range = 1.99–17.2	Range = 23–38	Range = 1.99–38.1
**Weight (kg)**	Median = 18	Median = 62	Median = 22
	IQR = 13.2–41.7	IQR = 57–68.25	IQR = 14.1–54.0
	Range = 10.4–82.0	Range = 54–75	Range = 10.4–82.0
**Co-morbidities**			
Num. of patients	*n* = 10 (66.7%)	*n* = 3 (75%)	*n* = 13 68.4%
Num. per patient	Median = 2	Median = 2	Median = 2
	IQR = 1.3–2	IQR = 1.5–2.5	IQR = 1–2
	Range = 1–4	Range = 1–3	Range = 1–4
Dev. delay	*n* = 8 (53.3%)	*n* = 3 (75%)	*n* = 11 57.9%
**Epilepsy etiology**			
Genetic	*n* = 8 (53.3%)	*n* = 2 (50%)	*n* = 10 (52.6%)
Unknown	*n* = 6 (40.0%)	*n* = 2 (50%)	*n* = 8 (42.1%)
Structural	*n* = 1 (6.7%)	*n* = 0 (0%)	*n* = 1 (5.3%)
**Seizure treatments**			
Ketogenic diet	*n* = 3 (20.0%)	*n* = 0 (0%)	*n* = 3 (15.8%)
VNS	*n* = 2 (13.3%)	*n* = 0 (0%)	*n* = 2 (10.5%)
DBS	*n* = 1 (6.7%)	*n* = 0 (0%)	*n* = 1 (5.3%)
**Pre-CBPM ASMs trialed**			
Frequency	*n* = 15 (100%)	*n* = 4 (100%)	*n* = 19 (100%)
Number per patient	Median = 3	Median = 5	Median = 3
	IQR = 2.5–3	IQR = 5–5	IQR = 3–5
	Range = 1–6	Range = 5–5	Range = 2–6
**Concomitant to ASMs**			
Frequency	*n* = 14 (93.3%)	4 (100%)	18 (94.7%)
Number per patient	Median = 2	Median = 2.5	Median = 2
	IQR = 1–3	IQR = 1.8–3.0	IQR = 1–3
	Range = 1–4	Range = 1–3	Range = 1–4
**Seizures pre-CBPM**			
Frequency/month	Median = 28	Median = 2	Median = 6.5
	IQR = 3–122	IQR = 1.88–2.25	IQR = 2–69.5
	Range = 0.5–12,300	Range = 1.5–3	Range = 0.5–12,300
**Seizure types**			
Number per patient	Median = 2	Median = 1.5	Median = 2
	IQR = 2–2	IQR = 1–2.25	IQR = 1.5–2
	Range = 1–4	Range = 1–3	Range = 1–4

### Seizure freedom

#### Seizure free periods

Table 3 provides summary information on the cohort’s cumulative SF experience. All patients experienced the cessation of all seizure types. The median cumulative duration of reported SF was 245 days (range 90–1,694). These were split between different continuous SF periods, though most patients reported experiencing just one continuous SF period (*n* = 14), with the median amount of SF periods per patient being 1 (range 1–3). Five pediatric patients had at least one continuous SF period lasting ≥ 1 year, representing 4.0% of the pediatric DRE cohort. As per each patient’s last check-in with their neurologist, 26% (*n* = 5) remain seizure-free as of October 2024.

**TABLE 3:** Data on cumulative SF experience.

Overall statistics	Pediatric (*n* = 15)	Adult (*n* = 4)	Total (*N* = 19)
**Rate of SF from DRE group**			
≥ 90 days SF	*n* = 15/124 (12.1%)	*n* = 4/50 (8%)	*n* = 19/174 (10.9%)
≥ 365 days SF	*n* = 5/124 (4.0%)	*n* = 0 (0%)	*n* = 5/174 (2.9%)
**Currently SF (Oct 2024)**	*n* = 5 (33.3%)	*n* = 0 (0%)	*n* = 5/19 (26.3%)
**Cumulative SF duration (days)**	Median = 333	Median = 195.5	Median = 245
	IQR = 129.5–536	IQR = 172–219.5	IQR = 142–406.5
	Range = 90–1,694	Range = 148–245	Range = 90–1,694
**Percent time seizure free while on CBPMs**	Median = 55.2%	Median = 61.6%	Median = 50.0%
	IQR = 23.3–86.4	IQR = 49.2–78.4	IQR = 35.5–86.0
	Range = 8.5–94.77	Range = 46.9–94.3	Range = 8.5–94.7
**Number of SF periods/patient**	Median = 1	Median = 1	Median = 1
	IQR = 1–2	IQR = 1–1	IQR = 1–1
	Range = 1–3	Range = 1–1	Range = 1–3
**Quality of life**			
Improved	*n* = 13 (86.7%)	*n* = 4 (100%)	*n* = 17 (89.5%)
No change	*n* = 2 (13.3%)	*n* = 0 (0%)	*n* = 2 (13.3%)
**CBPM adverse effects**			
Reported	*n* = 8 (53.3%)	*n* = 2 (50%)	*n* = 10 (52.6%)
**Common AEs**			
Drowsiness	*n* = 5 (33.3%)	*n* = 0 (0%)	*n* = 5 (26.3%)
Reduced appetite	*n* = 2 (13.3%)	*n* = 1 (25%)	*n* = 3 (15.8%)
Fatigue	*n* = 2 (13.3%)	*n* = 1 (25%)	*n* = 3 (15.8%)
Behavioral events	*n* = 2 (13.3%)	*n* = 0 (0%)	*n* = 2 (10.5%)
Poor sleep	*n* = 1 (6.7%)	*n* = 1 (25%)	*n* = 2 (10.5%)
GI issues	*n* = 2 (13.3%)	*n* = 0 (0%)	*n* = 2 (10.5%)
Vomiting	*n* = 1 (6.7%)	*n* = 0 (0%)	*n* = 1 (5.3%)
**Complete ASM weaning**			
All ASMs	*n* = 3 (33.3%)	*n* = 0	*n* = 3 (15.8%)

Supplementary Table 3 summarizes data on each continuous SF period, while a patient-specific breakdown of CBPM and SF periods can be found on Table 4. At the beginning of their treatments, 9 patients were on a CBD-only treatment, while the remaining 10 patients were using a combined CBD and THC treatment. Three of the four adults patients (75%) started on CBD only.

**TABLE 4:** Patient breakdown of each SF period and associated CBPM regimens and dosing.

Ptx #	# SF periods	Achieving SF 1 from CBPM start	CBPM regimen at SF	CBD dose (mg/kg/day)	THC dose (mg/kg/day)	SF Duration (days)	Breakthrough seizures context	Total duration of SF	MC side effects	Currently SF? (as of Oct 2024)
**Pediatric**										
1	3	No change	CBD+THC	10	0.7	230	Illness; no clear triggers	562	Drowsiness, fatigue	No
			CBD+THC	17	0.45	153				
			CBD+THC	21.25	0.45	179 (BT seizure)				
2	1	No change	CBD-only	8.25	0	401 (last FU)	N/A	401	Poor sleep, behavior, reduced appetite	Yes
3	1	Reduced CBD/THC	CBD+THC	10	0.5	180 (BT seizure)	No clear triggers	180	Drowsiness	No
4	1	No change	CBD+THC	4	0.19	412 (last FU)	No clear triggers	412	Drowsiness	Unknown (lost to FU)
5	1	No change	CBD-only	15	0	136 (BT seizure)	Illness	136	None	No
7	3	Added THC; increased CBD	CBD+THC	8.55	0.11	384	Illness; weight gain	1,694	None	Yes
			CBD+THC	5.33	0.2	436				
			CBD+THC	8	0.48	874 (last FU)				
8	1	No change	CBD+THC	6.6	0.22	338 (BT seizure)	No clear triggers	338	Fatigue, reduced appetite, GI issues	No
11	1	Reduced CBD	CBD-only	4.3	0	259 (last FU)	CBPM weaning	259	None	Yes
12	1	No change	CBD+THC	10	0.5	90 (BT seizure)	No clear triggers	90	None	No
13	1	No change	CBD+THC	2.6	0.09	123 (last FU)	N/A	123	None	Yes
14	2	Added THC; increased CBD	CBD+THC	8.64	0.4	390	Illness; no clear trigger	510	Drowsiness	No
			CBD+THC	8	0.49	120 (BT seizure)				
15	2	Added THC; increased CBD	CBD+THC	2.2	0.9	183	No Data	732	None	Unknown (lost to FU in 2020)
			CBD+THC	2.64	0.09	549 (last FU)				
17	1	No change	CBD+THC	43	0.62	120 (BT seizure)	No clear triggers	120	GI issues, behavioral events	No
18	1	Reduced CBD/THC	CBD+THC	0.71	0.02	333 (last FU)	N/A	333	None	Yes
19	1	No change	CBD+THC	12.5	0.86	101 (BT seizure)	Illness	101	Drowsiness, vomiting	No
**Adults**										
6	1	No change	CBD+THC	1.97	0.1	180 (BT seizure)	Illness	180	None	No
9	1	Reduced CBD	CBD-only	6.8	0	211 (BT seizure)	No clear triggers	211	None	No
10	1	No change	CBD-only	4.3	0	148 (last FU)	N/A	148	Poor sleep, fatigue	No
16	1	Added THC; increased CBD	CBD+THC	3.6	0.03	245 (last FU)	N/A	245	Appetite	Unknown (lost to FU in 2020)

#### Achieving seizure freedom

In the transition between starting CBPMs and achieving the first continuous SF period, 11 patients did not change their treatment or target dosages, as they became seizure-free when they started CBPMs. For instance, patient 7 became seizure-free after one week of CBPM treatment and was no longer exhibiting ataxia. Four patients added THC to their CBD-only treatment and adjusted their CBD dose. This included patient 17, who adjusted their CBD dose and added THC. Patients 12 and 16 added THC to address their aggressive behavior and sleep/anxiety, respectively. Other changes involved adjustments of dosages in both CBD and THC. For instance, patient 13 switched to a different CBD product (i.e., a 1:20 CBD oil to another company’s 1:30 CBD oil). Before achieving SF, Patient 11 had to wean CBPMs briefly due to product unavailability, which led to a significant worsening of seizure frequency. Once the product became available again, patient 11 achieved SF.

#### CBD and THC dosing associated with SF achievement

Looking at patients’ first continuous SF period (Table 4 and Supplementary Table 3), the median duration of SF was 211 days (range 90–412). Five patients (26.3%) were on CBD only, while 14 (73.7%) were on a combination of CBD and THC. The associated median CBD doses for CBD only and CBD+THC regimens were 6.8 (range 4.3–15) and 7.58 (range 0.71–43) mg/kg/day, respectively. The median THC dose during this first SF period was 0.31 mg/kg/day (range 0.02–0.9). Supplementary Table 3 presents these doses split into the adult and pediatric cohorts.

#### Breakthrough seizures

Of the patients with multiple SF periods, the median time between the first and second was 106.5 days (range 20–174). Most patients experienced breakthrough seizures (*n* = 13) (Table 4 and Supplementary Table 3) during their first SF period. Though 6 of these had no clear triggers, multiple events were associated with breakthrough seizures onset, including illness (*n* = 5), ASM weaning (*n* = 1), CBPM weaning (*n* = 1), and weight gain (*n* = 1). Patients 1 and 14 both noted illness associated with breakthrough seizures, but these were not identified as a definitive trigger in those individuals. Patient 7 had breakthrough seizures following weight gain and intercurrent illnesses. In the transition between the first and second SF periods, CBPM products and/or dosages of CBD and/or THC were adjusted. This includes patient 1, who switched from taking full spectrum CBPMs (Shubie Oil and Banook Oil) to a combination of separate products of purified CBD (Rho Phyto) and THC-rich extract (THC Reign Drops) to achieve their second SF period following breakthrough seizures (Supplementary Table 4). Patient 7, who experienced breakthrough seizures in the context of illness and weight gain, added THC at night (Tilray THC Oil), resulting in their second SF period, which has lasted for > 2 years.

#### Second SF period following breakthrough seizures

During the patients’ second SF period, no patients were using CBD-only CBPMs (Supplementary Table 3). All of these patients were pediatric. The median duration of the second SF period was 294.5 days (range 120–592). The associated median CBD and THC doses were 6.67 (range 2.64–17) and 0.33 (range 0.09–0.49) mg/kg/day. During their second SF period, patient 15 weaned CBPMs and all ASMs due to the complete remission of seizures. They remained seizure-free as of October 2023. Though patient 4 experienced a second SF period, this was only after adjusting both CBPM and oxcarbazepine dosing, so it was not included in this study due to this study’s fourth exclusion criterion. Extended data on patients’ third continuous SF period can be found in Supplementary Table 3.

### Secondary outcomes

#### Seizure frequency when not seizure-free

The median monthly seizure frequency prior to CBPM treatment was 6.5 (range 0.5–12,300) (Table 2). This decreased to 2 (range 0.25–990) when patients were on CBPMs but not in their SF period (i.e., during their breakthrough seizures or post-SF periods). This was a 69.2% reduction in monthly seizure frequency. Eleven patients (57.9%) experienced an interval breakthrough seizures period during CBPM treatment, of which 4 (21.1%) re-established a second SF period (Table 4 and Supplementary Table 3) using CBPMs.

#### Quality of life

Seventeen (89.5%) patients reported improvements in QoL; 2 (10.5%) reported no change (Table 3). Ten (52.6%) patients reported an AE when on CBPMs. Five of these patients reported AEs only at the initialization of CBPM treatment. The most commonly reported AEs were sleepiness/drowsiness (*n* = 5), reduced appetite (*n* = 3), and increased fatigue (*n* = 3). Patients 2 and 10 reported sleep issues: both reported sleep maintenance issues, and one reported issues initiating sleep as well (Table 3). The AEs are summarized in Table 3. No severe AEs were reported in this cohort; CBPMs were generally well-tolerated.

#### Concomitant ASMs

Most patients were taking concomitant clobazam or VPA while seizure-free and did not completely wean off concomitant ASMs. Three (15.8%) patients of the cohort completely weaned off all concomitant ASMs while taking CBPMs. These were patient 2 (clobazam and topiramate); patient 5 (clobazam); and patient 7 (topiramate). Patient 15 weaned off just one of their ASMs (clobazam). Though patient 4 did wean oxcarbazepine, they experienced a BT seizure, and had to re-administer oxcarbazepine.

## Discussion

In this RWE case series of 19 patients with DRE who underwent periods of SF (≥ 90 days) during CBPM treatment, we provide practical information on SF in the context of CBPMs. Unlike past trials reporting SF after treatment with pharmaceutical-grade CBD (Devinsky et al., 2011; Devinsky et al., 2016; Devinsky et al., 2018a; Devinsky et al., 2019; Thiele et al., 2019; Patel et al., 2021; Scheffer et al., 2021; Thiele et al., 2021), our SF population was not limited to certain syndromes (i.e., Lennox-Gastaut, Dravet). This indicates CBPM efficacy in other syndromes and is consistent with past research that has shown similarly efficacious effects of CBPMs in other DRE subtypes (Stockings et al., 2018; Espinosa-Jovel et al., 2023; Kühne et al., 2023). Though we did not assess 50% seizure response rates, which has previously been reported to be around 35%–63% (Hausman-Kedem et al., 2018; McCoy et al., 2018; Devinsky et al., 2019; Devinsky et al., 2020; Lattanzi et al., 2020a; Thiele et al., 2022; Espinosa-Jovel et al., 2023), our data showed that ∼4% of our pediatric DRE population achieved at least 1 year of SF.

Patients spent approximately 50% of their total CBPM treatment duration seizure-free, from a median pre-CBPM seizure rate of 6.5 seizures per month. The median duration of the first SF period was 211 days, while the cohort’s median total SF duration was 245 days, ranging from 90 to 1,694 days, spread over 1 to 3 SF periods. These findings set expectations for patients, caregivers and clinicians providing a foundation for future research to explore and detail CBPM-related seizure-free periods more comprehensively. Given the well-known high costs associated with managing seizures in DRE (Widjaja et al., 2021), and resultant SF within our study population, it would be beneficial to assess the economics of a CBPM-related SF period.

Taken together, with similar findings reported in past research (Devinsky et al., 2011; French et al., 2017; Devinsky et al., 2018b; Chakraborty and Hocker, 2019; Devinsky et al., 2019; Thiele et al., 2019; Thiele et al., 2022; Wu et al., 2022), the outcomes of CBPM treatment in our cohort align with the priorities/goals of patients and caretakers, including SF, improved QoL, low AE impact, and complete ASM weaning. Seizure freedom and its associated QoL improvements (Jacoby et al., 2009; Ring et al., 2019) are recognized as the main goals of epilepsy treatment among patients, families, and treatment guidelines (Josephson et al., 2017; Halford and Edwards, 2020). Patients and caretakers also prioritize low AE profiles in ASM trials (Jacoby et al., 2009). These priorities, coupled with our results, highlight the need to further assess the nature and long-term effects of CBPM-related seizure freedom in more rigorous studies. This would help improve CBPM access as a DRE treatment and support the more accurate disclosure of current, established CBPM-related research findings by epileptologists to patients with DRE and their families.

Our cohort’s SF periods were substantial, considering the patients’ DRE statuses, and the difficulty individuals with DRE have in achieving SF. Previous research shows that after trialing 3 ASMs, patients have at most a 1% likelihood of achieving SF as defined by the ILAE (Kwan et al., 2010) (i.e., at least a year without seizures) when trialing further regimens (Chen et al., 2018). Despite the introduction of more than a dozen new ASMs between 2000 and 2018, these likelihoods and SF rates have remained unchanged (Kwan and Brodie Martin, 2000; Chen et al., 2018). In contrast, 4% of our pediatric DRE population that was treated with CBPMs—having tried a median of 3 ASMs prior to CBPM treatment—achieved 1 year of SF. This is a significant improvement from a 1% likelihood of achieving SF with other ASMs and aligns with past CBPM RCTs (Table 1), which reported that 5% of DRE patients treated with CBPMs undergo significant periods of SF.

Given the consistency across independent studies of varying rigor—from observational trials to high-quality randomized control trials—rates of SF freedom reported with CBPMs and observed in the analysis of our data set present a clinical outcome that should be actively considered and evaluated. CBPMs modulate the endocannabinoid system, thereby acting via a unique mechanism in comparison to conventional ASMs. CBD and THC have distinct pharmacological targets on the nervous system. While CBD has a relatively low affinity (4,350 μM Ki) at the CB1 receptor (De Petrocellis et al., 2011), it inhibits fatty acid amide hydrolase (FAAH) enzyme activity, which hydrolyzes the endocannabinoid anandamide, thereby enhancing its signaling. Multi-modal mechanisms described in the literature provide a theoretical and experimentally demonstrated basis for stabilizing neuronal excitatory:inhibitory balance via interactions with the 5-HT1A, 5HT3, TRPV1 and GRP55 receptors in addition to the ENT-1 adenosine transporter (Townsend et al., 2002, Gray and Whalley, 2020). Pre-clinical support has been demonstrated in acute mouse brain slices that inhibiting the 5-HT1A receptor blocks CBD’s anti-convulsant effects (Javadzadeh et al., 2024). THC, on the other hand, is a partial agonist of both the CB1 and CB2 receptors. Cumulatively, CBD and THC have diverse pharmacological effects modulating neurotransmission and inflammation that provide a theoretical neurobiological basis as to why a significant and consistent minority of patients with DRE appear to robustly respond to CBPMs. Though further rigorous research is needed to ascertain the mechanisms of action in CBPM-related seizure freedom and the SF rate in DRE populations, our data supports improving CBPM accessibility to those with DRE. This paper also supports pursuing placebo-controlled studies that assess whether CBPMs should be considered a prioritized pharmacological treatment for DRE alongside epilepsy surgery referral, given the SF findings from this study and previous research. Future research should also actively compare seizure freedom and 50% seizure response rates between CBPMs and ASMs in DRE, to further define CBPMs role in DRE treatment.

The findings of this study and the priorities of PWE also highlight the greater issue regarding the importance of using SF data to assess ASMs. Clinical and research-based assessments of ASMs focus on seizure frequency reductions without reporting SF data (Halford and Edwards, 2020). Seizure frequency reductions certainly have value; however, focus should not be diverted from SF, especially considering that non-seizure-free reductions in seizure frequency are associated with reduced QoL, increased social isolation and stigmatization, increased self-reported mood disorders, greater psychological distress, and lower employment rates than SF (Jacoby et al., 2009; Josephson et al., 2017; Ring et al., 2019). Shifting a greater degree of focus to SF in ASM assessments in DRE would align with patient outcomes and desires, and could facilitate a reframing of ASM testing that previous researchers have called for due to the lack of change in DRE SF rates from ASMs over the past 20 years (Chen et al., 2018; Halford and Edwards, 2020).

### Study limitations

The retrospective case series study design risks the inclusion of selection bias and potential confounds. These, combined with the lack of randomization, placebo comparison, and low sample size, prevent us from drawing causal conclusions. For example, due to the design of the study, we were unable to assess and report an accurate 50% seizure responder rate in our total population. The cohort was also heterogeneous in seizure types, etiology, age, treatment protocols, and medical history. Our use of patient chart data also resulted in heterogeneous entries, missing data, and reliance on patient reports. These hinder the ability to draw comparisons between patients, especially since patient reports on seizure tracking may be biased (e.g., placebo effect after CBPM was initiated) and inaccurate. This is further limited by the absence of EEG data in our analyses. Though EEGs were used to confirm baseline diagnoses and seizure types, these were unavailable for analysis, and were not performed during or after CBMP treatment. Thus, we cannot ascertain if patients were experiencing subclinical seizures. However, during SF periods, no patients/caregivers reported clinical signs of worrisome subclinical events (e.g., overly fatigued, unwell, not at baseline level of health). Despite the limitations, it is important to note that the authors have reported a dichotomous variable (i.e., complete absence of seizures) and, therefore, these limitations may not be as impactful in a similar study solely evaluating seizure reduction in a DRE population.

## Conclusion

This study reports RWE from 19 patients with DRE who experienced SF due to CBPM therapy. The SF rates observed in this study complement existing literature that reports rates of at least 4%, higher than the 1% observed with established ASMs.

CBPMs are pharmacologically distinct from conventional ASMs, acting through unique mechanisms via modulation of the ECS. Therefore, patients whose epilepsies respond particularly well to CBPMs may represent a distinct cohort with shared neurobiological and clinical features. Given the significant burden that ongoing seizures pose on morbidity, mortality, QoL, and healthcare costs, the authors call on the epilepsy research community to prioritize the identification of this population’s shared characteristics. Focusing on the identification of their chemical and genetic biomarkers may translate clinically in the guidance of treatment choice and the prioritization of CBPMs in treatment pathways for DRE. Given the comparative SF data in the published literature, future double blind, placebo-controlled studies should also assess whether CBPMs should be prioritized as first-line medical therapy for DRE cases that lack established, evidence-based treatment options.

Finally, the RWE presented in this study supports the need for greater accessibility to CBPMs, a comparative economic analysis of the costs associated with DRE patients not treated with CBPMs, and the inclusion of SF data in future CBPM epilepsy trials.

## Data Availability

The original contributions presented in this study are included in this article/Supplementary material, further inquiries can be directed to the corresponding author.

## Supplementary material

Supplementary Figure 1 Standardized CBPM dosing protocol designed by ECL and the NTN neurological care team.
