# Supplementary material for: 19 patients report seizure freedom with medical cannabis oil treatment for drug-resistant epilepsy: a case series
Source: Front Neurosci. 2025 May 19;19:1570531. doi: 10.3389/fnins.2025.1570531 (PMC12127399; doi:10.3389/fnins.2025.1570531)
Supplement: Supplementary file 1 [file Data_Sheet_1.docx]

**Table S1. Full demographic details of each patient.**

Abbreviations: GTC = generalized tonic-clonic seizure; ADHD = attention deficit hyperactivity disorder; ASD = autism spectrum disorder; GDD = global developmental delay.

| **Patient** | **DOB** | **Age (first SF)** | **Sex** | **Development Delay** | **Co-Morbidities**  Number  (specifics) | **Weight (kg)** | **Age (Seizure Onset)** | **Epilepsy Etiology** | **Seizure Types** | **Monthly seizure frequency before CBMP** |
| --- | --- | --- | --- | --- | --- | --- | --- | --- | --- | --- |
| 1 | 31-Jul-17 | 2.68  Pediatric | F | Y | 2  (Transaminitis; ASD) | 12.7 | 0.33 | Genetic (SCN1A) | Focal seizures (secondary generalization); Absence | 0.5 |
| 2 | 07-Mar-18 | 4.24  Pediatric | M | N | 2  (ADHD; ASD) | 17.3 | 3 | Unknown | GTC; Absence | 28 |
| 3 | 12-Nov-18 | 2.14  Pediatric | F | N | 1  (ASD) | 12.2 | 4 | Genetic (SCN1A) | GTC; Focal Clonic; Absence | 24 |
| 4 | 01-Mar-03 | 17.18  Pediatric | F | Y | 0 | 82 | 1 | Genetic (Tuberous sclerosis complex) | GTC | 1.5 |
| 5 | 23-Sep-20 | 1.99  Pediatric | F | Y | 2  (ASD; GDD) | 10.4 | 0.25 | Genetic (SCN1A) | Focal; Absence | 3 |
| 6 | 03-Oct-94 | 25.35  Adult | F | Y | 3  (diabetes; GDD; previous spongy cardiomyopathy) | 58 | 5 | Unknown etiology | Focal with impaired awareness | 2 |
| 7 | 05-Nov-15 | 3.78  Pediatric | F | N | 0 | 12.8 | 2 | Unknown etiology | GTC; Left focal tonic-clonic | 30 |
| 8 | 23-May-08 | 13  Pediatric | M | N | 1  (ADHD) | 45.4 | 9 | Unknown etiology | GTC | 3 |
| 9 | 15-Oct-96 | 25.90  Adult | M | N | 0 | 66 | 23 | Unknown etiology | Focal (secondary generalization);  Focal with loss of awareness | 2 |
| 10 | 29-Oct-84 | 38.12  Adult | F | Y | 2  (GDD; hypotonia) | 75 | 0.54 | Genetic (SCNA1) | GTC | 3 |
| 11 | 08-Sep-12 | 10.07  Pediatric | F | N | 1  ASD | 38 | 6 | Unknown etiology | GTC; Myoclonus | 12,300 |
| 12 | 20-Aug-12 | 7.58  Pediatric | F | Y | 2  (ASD; GDD) | 22 | 1.83 | Genetic (ANKRD11) | Drop; GTC; Myoclonic jerks Absence | 94 |
| 13 | 17-Aug-16 | 4.72  Pediatric | M | Y | 2  (GDD; non-verbal) | 22 | 1 | Genetic (Angelman syndrome - deletion chromosome 15) | GTC; Absence | 390 |
| 14 | 23-Mar-08 | 11.36  Pediatric | F | N | 0 | 54 | 0 | Unknown etiology | Focal left sensorimotor; GTC | 150 |
| 15 | 20-May-12 | 5.78  Pediatric | M | N | 0 | 18 | 2 | Unknown etiology | Night time events | 6.5 |
| 16 | 29-Sep-99 | 20.52  Adult | F | Y | 0 | 54 | 0.7 | Genetic (Tuberous Sclerosis Complex) | GTC; Focal seizures with impaired awareness; Absence | 1.5 |
| 17 | 04-May-17 | 5.08  Pediatric | M | Y | 4  (Allergies; polymicrogyria; hypotonic cerebral palsy; dystonia) | 13.6 | 1 | Structural (polymicrogyria) | Focal; Alternating hemiplegia (non-epileptiform) | 8.3 |
| 18 | 21-Dec-17 | 4.03  Pediatric | M | Y | 0 | 14.6 | 2 | Genetic (Doose syndrome) | GTC; Head drops | 465 |
| 19 | 26-Jul-03 | 16.44  Pediatric | M | Y | 3  (GDD; bilateral pes planus; intellectual disability) | 48.5 | 4 | Unknown etiology | Drop; GTC; Myoclonic; Absence; Tonic | 45 |
