# Supplementary material for: 19 patients report seizure freedom with medical cannabis oil treatment for drug-resistant epilepsy: a case series
Source: Front Neurosci. 2025 May 19;19:1570531. doi: 10.3389/fnins.2025.1570531 (PMC12127399; doi:10.3389/fnins.2025.1570531)
Supplement: Supplementary file 2 [file Data_Sheet_2.docx]

**Supplemental Table S2. Treatments for seizure management before, after, or concomitant with CBPMs.**

| **Ptx #** | **Ketogenic Diet** | **VNS** | **DBS** | **# Pre-CBPM ASMs** | **Pre-CBPM ASMs trialled** | **# ASMs Concomitant** | **Concomitant ASMs** | **Amount ASM after MC** |
| --- | --- | --- | --- | --- | --- | --- | --- | --- |
| 1 | N | N | N | 3 | Stiripentol, Valproic acid, Levetiracetam, Clobazam | 3 | Valproic acid, Clobazam, Levetiracetam | N/A |
| 2 | N | N | N | 5 | Levetiracetam, Clobazam, Topiramate, Valproate, Brivaracetam | 3 | Clobazam, Topiramate | N/A |
| 3 | Y | Y | N | 3 | Clobazam, Levetiracetam, Valproic acid | 3 | Levetiracetam, Clobazam, Valproic acid | N/A |
| 4 | Y | N | N | 3 | Clobazam, Topiramate, Oxcarbazepine | 1 | Oxcarbazepine | N/A |
| 5 | N | N | N | 2 | Levetiracetam, Clobazam | 1 | Clobazam | N/A |
| 6 | N | N | N | 5 | Prednisone, Gabapentin, Clobazam, Lacosamide, Levetiracetam | 3 | Clobazam, Lacosamide, Levetiracetam | 3 |
| 7 | N | N | N | 3 | Levetiracetam, Valproic acid, Topiramate | 1 | Topiramate | N/A |
| 8 | N | N | N | 3 | Levetiracetam, Clobazam, Aptiom | 1 | Aptiom | N/A |
| 9 | N | N | N | 5 | Oxcarbazepine, Dilantin, Brivaracetam, Lamotrigine, Clobazam | 2 | Brivaracetam, Clobazam | N/A |
| 10 | N | N | N | 5 | Clobazam, Levetiracetam, Divalproex Sodium, Phenobarbital, Dilantin | 3 | Divalproex Sodium, Levetiracetam, Clobazam | N/A |
| 11 | N | N | N | 3 | Valproic acid, Clobazam, Topiramate | 2 | Valproic acid, Clobazam | N/A |
| 12 | N | Y | Y | 6 | Levetiracetam, VPA, Lamotrigine, Topiramate, Clobazam, Rufinamide | 2 | Clobazam, Rufinamide | N/A |
| 13 | Y | N | N | 2 | Clobazam, Ethosuximide | 1 | Clobazam | N/A |
| 14 | N | N | N | 6 | Levetiracetam, Perampanel, Carbamazepine, Clobazam, Lamotrigine, Divalproex | 4 | Lamotrigine, Clobazam, Brivaracetam, Lacosamide | N/A |
| 15 | N | N | N | 2 | Carbamazepine, Clobazam |  |  | N/A |
| 16 | N | N | N | 5 | Gabapentin, Vigabatrin, Topiramate, Divalproex, Valproate | 1 | Divalproex | N/A |
| 17 | N | N | N | 3 | Valproic acid, Gabapentin, Flunarizine | 4 | Valproic acid, Gabapentin, Topiramate, Flunarizine | N/A |
| 18 | N | N | N | 3 | Clobazam, VPA, Levetiracetam | 3 | Valproic acid, Levetiracetam, Ethosuximide | N/A |
| 19 | N | N | N | 2 | Valproic acid, Lacosamide | 2 | Valproic acid, Clobazam | N/A |
