# Supplementary material for: 19 patients report seizure freedom with medical cannabis oil treatment for drug-resistant epilepsy: a case series
Source: Front Neurosci. 2025 May 19;19:1570531. doi: 10.3389/fnins.2025.1570531 (PMC12127399; doi:10.3389/fnins.2025.1570531)
Supplement: Supplementary file 3 [file Data_Sheet_3.docx]

**Table S3.** Data on first continuous substantial SF period.

| Summary data (1^st^ SF periods) | Pediatric (< 18 yo) (n=15) | Adult (n=4) (≥ 18 yo) | Total (N=19) |
| --- | --- | --- | --- |
| Pre-SF | | | |
| CBPM regimen pre-SF  CBD+THC  CBD-only  CBPM dosing pre-SF (mg/kg/day)  CBD (CBD-only)  CBD (CBD+THC)  THC (CBD+THC) | n = 9 (60.0%)  n = 6 (40.0%)  Median = 7.40  IQR = 5.39-8.56  Range = 1.25-15.00  Median = 9.45  IQR = 3.90-12.41  Range = 2.59-42.99  Median = 0.50  IQR = 0.20-0.68  Range = 0.05-0.85 | n = 1 (25%)  n = 3 (75%)  Median = 4.33  IQR = 3.12-6.28  Range = 1.89-8.22  Median = 1.97  IQR & range = N/A (n=1)  Median = 0.098  IQR & range = N/A (n=1) | n = 10 (52.6%)  n = 9 (47.4%)  Median = 6.58  IQR = 4.33-8.24  Range = 1.25-15.00  Median = 8.03  IQR = 3.03-11.81  Range = 1.97-42.99  Median = 0.36  IQR = 0.122-0.67  Range = 0.05-0.85 |
| First Continuous SF Period | | | |
| Achieving 1^st^ SF  No change  Added THC + increased CBD  Reduced THC and CBD  Reduced CBD | n = 9 (60.0%)  n = 3 (20.0%)  n = 2 (13.3%)  n = 1 (6.7%) | n = 2 (50%)  n = 1 (25%)  n = 0 (0%)  n = 1 (25%) | n = 11 (57.9%)  n = 4 (21.1%)  n = 2 (13.3%)  n = 2 (13.3%) |
| CBPM regimen at 1^st^ SF  CBD-only  CBD+THC  CBPM dosing at 1^st^ SF (mg/kg/day)  CBD (CBD-only)  CBD (CBD+THC)  THC (CBD+THC) | n = 3 (20.0%)  n = 12 (80.0%)  Median = 8.25  IQR = 6.28-11.63  Range = 4.3-15  Median = 8.60  IQR = 3.65-10.0  Range = 0.71-43.0  Median = 0.45  IQR = 0.17-0.64  Range = 0.02-0.90 | n = 2 (50%)  n = 2 (50%)  Median = 5.55  IQR = 4.93-6.18  Range = 4.3-6.8  Median = 2.79  IQR = 2.38-3.19  Range = 1.97-3.6  Median = 0.065  IQR = 0.048-0.083  Range = 0.03-0.1 | n = 5 (26.3%)  n = 14 (73.7%)  Median = 6.8  IQR = 4.3-8.25  Range = 4.3-15  Median = 7.58  IQR = 2.85-10  Range = 0.71-43  Median = 0.31  IQR = 0.10-0.59  Range = 0.02-0.9 |
| 1^st^ SF period duration  Days | Median = 230  IQR = 129.5-361  Range = 90-412 | Median = 195.5  IQR = 172-219.5  Range = 148-245 | Median = 211  IQR = 142-335.5  Range = 90-412 |
| Breakthrough seizures  Reported  Common triggers  No clear triggers  Illness  Illness + weight gain  ASM weaning  CBPM weaning  N/A | n = 11 (57.9%)  n = 5 (33.3%)  n = 4 (26.7%)  n = 1 (6.7%)  n = 0 (0%)  n = 1 (6.7%)  n = 4 (26.7%) | n = 2 (50%)  n = 1 (25%)  n = 0 (0%)  n = 1 (25%)  n = 0 (0%)  n = 2 (50%) | n = 13 (68.4%)  n = 6 (31.6%)  n = 4 (21.1%)  n = 1 (5.3%)  n = 1 (5.3%)  n = 1 (5.3%)  n = 6 (31.6%) |
| Second Continuous SF Period | | | |
| Re-established 2^nd^ SF | 4 (26.7%) | n = 0 (0%) | 4 (21.1%) |
| CBPM regimen at 2^nd^ SF  CBD-only  CBD+THC  CBPM dosing at 2^nd^ SF (mg/kg/day)  CBD (CBD+THC)  THC (CBD+THC) | n = 0 (0%)  n = 4 (26.7%)  Median = 6.67  IQR = 4.67-10.25  Range = 2.64-17.00  Median = 0.33  IQR = 0.17-0.46  Range = 0.09-0.49 | n = 0 (0%)  n = 0 (0%)  N/A  N/A | n = 0 (0%)  n = 4 (21.1%)  Median = 6.67  IQR = 4.67-10.25  Range = 2.64-17.00  Median = 0.33  IQR = 0.17-0.46  Range = 0.09-0.49 |
| 2^nd^ SF period duration  Days | Median = 294.5  IQR = 144.8-464.3  Range = 120-529 | N/A | Median = 294.5  IQR = 144.8-464.3  Range = 120-529 |
| Third Continuous SF Period | | | |
| Re-established 3^rd^ SF | n = 2 (13.3%) | n = 0 (0%) | n = 2 (10.5%) |
| CBPM regimen at 3^rd^ SF  CBD-only  CBD+THC  CBPM dosing at 3^rd^ SF (mg/kg/day)  CBD (CBD+THC)  THC (CBD+THC) | n = 0 (0%)  n = 2 (13.3%)  Median = 14.63  Range = 8.00-21.25  Median = 0.47  Range = 0.45-0.48 | n = 0 (0%)  n = 0 (0%)  N/A  N/A | n = 0 (0%)  n = 2 (10.5%)  Median = 14.63  Range = 8-21.25  Median = 0.47  Range = 0.45-0.48 |
| 3^rd^ SF period duration  Days | Median = 526.5  Range = 179-874 | N/A | Median = 526.5  Range = 179-874 |
