# Supplementary material for: 19 patients report seizure freedom with medical cannabis oil treatment for drug-resistant epilepsy: a case series
Source: Front Neurosci. 2025 May 19;19:1570531. doi: 10.3389/fnins.2025.1570531 (PMC12127399; doi:10.3389/fnins.2025.1570531)
Supplement: Supplementary file 4 [file Data_Sheet_4.docx]

**Table S4. CBPM product names used by each patient.** The products included had to be used at least once during the duration of CBPM treatment and SF. Some patients used multiple products at once, while other patients switched between multiple products to just one product. We have indicated the THC:CBD ratios within each product.

| Patient ID | CBD-Isolate Product | Full Spectrum Product | THC-Isolate Product |
| --- | --- | --- | --- |
| 1 | Rho Phyto (0:50) | Shubie Oil (0.81:20.8) Banook Oil (20:1) | THC Reign Drops (30:0) |
| 2 | Rho Phyto |  |  |
| 3 |  | Shubie Oil |  |
| 4 | Rho Phyto | Shubie Oil  CBD Reign Drops (1:30) |  |
| 5 | Rho Phyto |  |  |
| 6 |  | Yellow Oil (1:20) |  |
| 7 |  | Tilray CBD oil (1:100) | Tilray THC (25:0) |
| 8 |  | CBD Reign Drops |  |
| 9 | Rho Phyto |  | Spectrum Red No. 1 (20:0) |
| 10 | Rho Phyto |  |  |
| 11 | Rho Phyto |  |  |
| 12 |  | Shubie Oil  CBD Reign Drops | THC Reign Drops |
| 13 |  | Aurora CanniMed CBD (1:20)  CDD Reign Drops |  |
| 14 |  | Avidekel, MedReLeaf (25:1) |  |
| 15 |  | Science Lab CBD tincture (n.d.) |  |
| 16 |  | Tilray CBD oil (2:100) | Tilray THC |
| 17 | Rho Phyto | Shubie Oil (1:20) | Spectrum Red No. 1 |
| 18 |  | Tilray CBD oil (2:100) |  |
| 19 | Midnight Oil (n.d.) | Medleaf oils (n.d.) |  |
