# Supplementary figures and images for: 19 patients report seizure freedom with medical cannabis oil treatment for drug-resistant epilepsy: a case series
Source: Front Neurosci. 2025 May 19;19:1570531. doi: 10.3389/fnins.2025.1570531 (PMC12127399; doi:10.3389/fnins.2025.1570531)

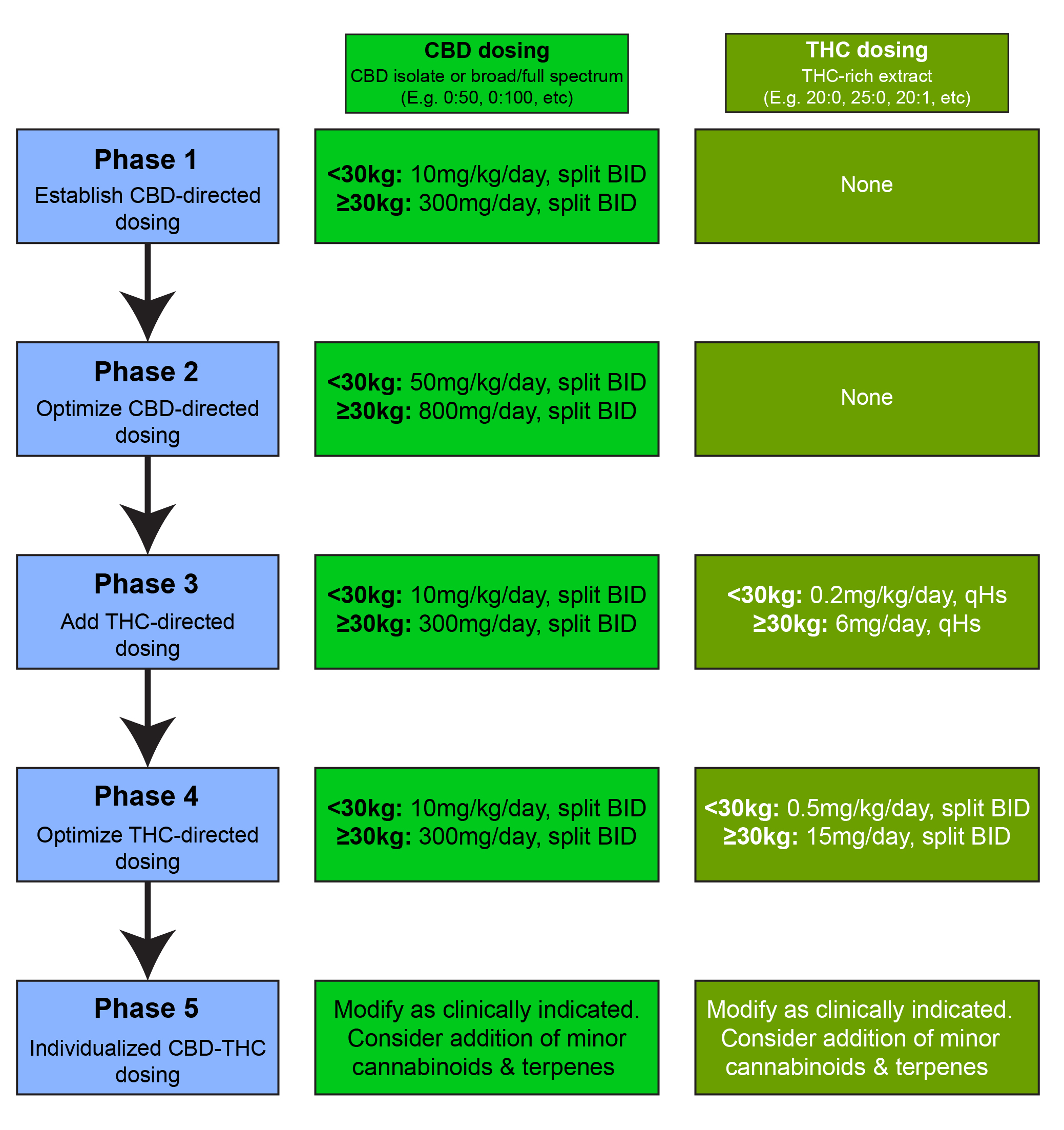

Supplement: Supplementary Figure 1 — Standardized CBPM dosing protocol designed by ECL and the NTN neurological care team. [file Image_1.jpeg]
